# Diffusion and Coalescence of Phosphorene Monovacancies Studied Using High-Dimensional Neural Network Potentials

**DOI:** 10.1021/acs.jpcc.3c05713

**Published:** 2023-12-05

**Authors:** Lukáš Kývala, Andrea Angeletti, Cesare Franchini, Christoph Dellago

**Affiliations:** † Faculty of Physics, University of Vienna, 1090 Vienna, Austria; ‡ Vienna Doctoral School in Physics, University of Vienna, 1090 Vienna, Austria; § Department of Physics and Astronomy, Università di Bologna, 40127 Bologna, Italy

## Abstract

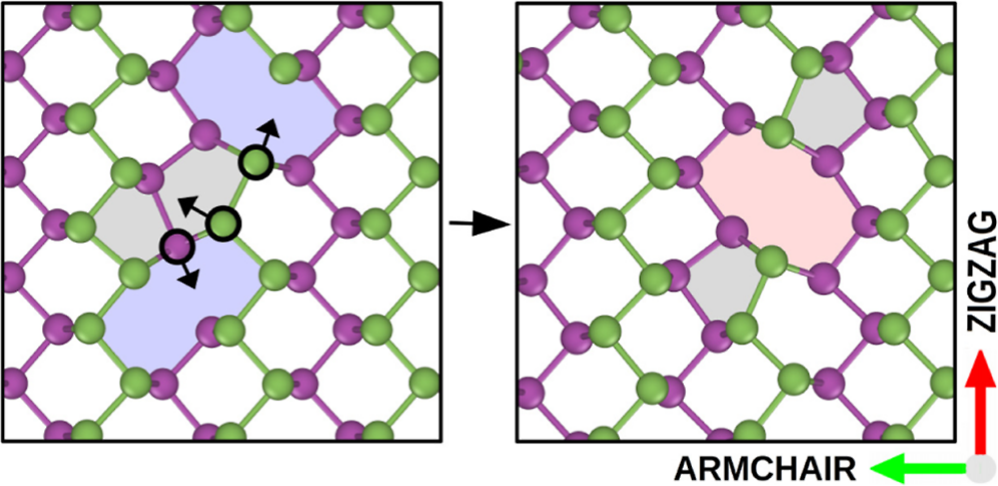

The properties of two-dimensional materials are strongly affected by defects that are often present in considerable numbers. In this study, we investigate the diffusion and coalescence of monovacancies in phosphorene using molecular dynamics (MD) simulations accelerated by high-dimensional neural network potentials. Trained and validated with reference data obtained with density functional theory (DFT), such surrogate models provide the accuracy of DFT at a much lower cost, enabling simulations on time scales that far exceed those of first-principles MD. Our microsecond long simulations reveal that monovacancies are highly mobile and move predominantly in the zigzag rather than armchair direction, consistent with the energy barriers of the underlying hopping mechanisms. In further simulations, we find that monovacancies merge into energetically more stable and less mobile divacancies following different routes that may involve metastable intermediates.

## Introduction

Two-dimensional (2D) materials are currently the subject of intense research because they show promise in a wide range of important applications, including electronics, valleytronics, catalysis, and biosensing.^1^ In particular, graphene has attracted much interest due to its large carrier mobility,^2^ but the lack of a band gap makes device applications difficult. Transition metal dichalcogenides (TMDs), which include MoS_2_ and WSe_2_, do feature band gaps within the range of 1.2–1.8 eV range.^1^ But as the valence and conduction bands of TMDs are made up of transition metal d-orbitals, their carrier mobility is a few orders of magnitude lower than that of graphene.^3^ In contrast, phosphorene, a 2D monolayer of black phosphorus, possesses a thickness-dependent electronic band gap (2.2 eV in the monolayer to 0.3 eV in bulk^4,5^), a high carrier mobility^6−9^ and in-plane anisotropy. Thanks to this unique combination of properties, phosphorene has been utilized in the fields of photocatalysis,^10,11^ semiconductors,^12,13^ rechargeable batteries,^14^ solar energy conversion,^15^ and gas sensors.^16^

Most properties and applications of materials are affected by the presence of defects, especially point defects.^17^ These imperfections give phosphorene its special properties, such as hole-doping,^18^ local magnetic moments,^18^ improved activity for hydrogen evolution reactions,^19^ enhanced ion-transport for alkali-ion batteries,^20^ and unique photoresponses for neuromorphic computing.^21,22^ Defects, however, can also act in undesirable ways that can lead to device failure. Therefore, understanding the formation and dynamics of defects is essential for the design of stable and functional phosphorene-based devices.

The simplest point defect in phosphorene, *i.e.*, the monovacancy, is the subject of this work. The localized magnetic moment originating from an unsaturated bond makes the monovacancy particularly intriguing. Despite being predicted to be the most common defect in phosphorene after electron and ion irradiation,^23,24^ a predominance of divacancies and tetravacancies has been observed in experimental studies.^25−27^ Only recent work claims to have captured individual monovacancies.^27^ The difficulty of observation can be attributed to the highly itinerant nature of monovacancies,^28^ making it difficult to identify them on the time scales accessible experimentally. Another reason may be that monovacancies coalesce rapidly into more stable divacancies. The detailed mechanism of monovacancy coalescence, however, has not yet been clarified.

In this work, we investigate the structure and kinetics of phosphorene monovacancies with extensive molecular dynamics (MD) simulations based on high-dimensional neural network potentials (HDNNP-MD).^29,30^ In this machine learning approach, the potential energy of the system is represented using artificial neural networks trained on energies and forces calculated using density functional theory (DFT), leading to a considerable increase in efficiency. From our simulations with an aggregate length of more than 1 μs, we identify the mechanism and rates of the hopping processes underlying defect diffusion, confirming the mechanism postulated previously, and investigate with the nudged elastic band method.^28^ We then investigate how two monovacancies approach each other and coalesce into a divacancy, finding that this process can occur following three different mechanisms depending on the orientation of the monovacancies and their direction of approach. Under certain conditions, a long-lived monovacancy pair forms, in which the monovacancies are arranged in a way such that all bonds are satisfied.

The remainder of the review is organized as follows. First, we outline the computational methods used in this work and provide technical details of the simulations. Subsequently, we present results obtained for the diffusion of a single vacancy, followed by a discussion of vacancy coalescence. Finally, we conclude our findings.

## Methods

In this section, we first discuss how the reference data are generated via first-principle calculations. We then describe the neural network model potential and explain how active learning was employed to generate additional reference data. Finally, we discuss neural network parametrizations and describe the MD setup.

### First-Principles Calculations

All reference data used for the neural network training were generated with first-principle calculations performed using the Vienna ab initio simulation package (VASP).^31,32^ The Perdew–Burke–Ernzerhof functional^33^ was employed with an energy cutoff of 600 eV. The optimized values of the lattice parameters for the pristine phosphorene structure are found to be 3.30 and 4.62 Å along the zigzag and armchair directions, respectively. The criterion for relaxation of the forces acting on each atom was set to 0.01 eV/Å. The supercell size chosen to simulate the system is 7 × 5 × 1, containing 140 atoms in the pristine structure with a vacuum region of 20 Å. The Brillouin zone was sampled using a 3 × 3 × 1 Γ-centered mesh. The ab initio MD simulations were performed with the Langevin thermostat^34,35^ in the *NPT* ensemble^36,37^ applying zero external pressure and 300 K with a reduced energy cutoff of 250 eV. By allowing the relaxation of the two primitive lattice vectors along the zigzag and armchair directions, we inferred the average size at room temperature of the supercell containing the defective structure with one monovacancy. The obtained values employed in all calculations are 3.32 and 4.56 Å, respectively. The climbing imaged nudged elastic band (ciNEB)^38^ calculations were performed using a relaxation criterion of 0.02 eV/Å for the forces acting on each image. The formation energy of a defect is defined as *E*_f_ = *E*_defective_ – *N*_p_ × *E*_p_, where *E*_defective_ is the energy of the structure with the defect, *N*_p_ is the number of atoms in the supercell, and *E*_p_ is the energy per phosphorus atom in the pristine configuration, *E*_p_ = *E*_pristine_/*N*_p_.

### Neural Network Potential

In this work, we employ the HDNNP-approach pioneered by Behler and Parrinello,^29^ in which the total potential energy of the systems is written as the sum of local, atom-centered contributions *E*_*i*_
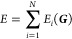
1where *N* denotes the number of atoms. Each contribution is encoded in an artificial neural network that depends on the local chemical environment described by a set of descriptors ***G***. These descriptors, also known as symmetry functions, are characteristic fingerprints of the chemical environment and are invariant with respect to translations, rotations, and permutations of atoms of the same species. As descriptors, we employ the so-called polynomial symmetry functions, a family of atom-centered symmetry functions based on polynomials with compact support.^39^ They provide more flexibility in the selection of angular symmetry functions while requiring fewer floating point operations for their evaluation than the original Behler–Parrinello symmetry functions.^29^ The forces acting on the atoms, necessary for the MD simulations, are given by the negative gradient of the potential energy, which is straightforward to calculate due to the simple analytical form of the neural network potential.

### Active Learning

To train our neural network potential, we use an active learning strategy called query by committee,^40^ which has proven useful for creating training sets for machine learning potentials.^41−44^ First, a few short ab initio MD simulations are performed, and configurations with energies and forces are collected from them. On this initial training set, several HDNNPs are trained with various training/validation splits and different initial conditions. The total energy  and the force component  acting on a atom *k* with respect to coordinate α are then calculated as averages over the committee
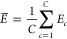
2
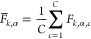
3here, *C* is the committee size, and *E*_*c*_ and *F*_*k*,α,*c*_ are the total energy and forces predicted by committee member *c*, respectively.

The committee disagreement is defined as the standard deviation of the committee’s predictions
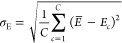
4
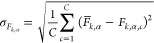
5

One expects that the predictions of different committee members are similar for configurations close to the training set and dissimilar elsewhere. Hence, the committee disagreement serves as a measure of the uncertainty of the neural network prediction.^42^ In the training phase, the neural network-accelerated MD is stopped when the committee disagreement exceeds a predetermined threshold. The simulation is then continued with DFT-MD, generating new configurations that are included in the training set. The neural networks are then retrained on this larger data set, and the MD simulation is resumed with the new neural networks and runs until the threshold is exceeded again. This procedure, repeated several times if necessary, makes sure that the training set contains all configurations that are required to accurately reproduce not only the perfect lattice and stable defects but also short-lived intermediate configurations without leading to excessive training set sizes. The uncertainty estimation based on the committee approach was also used during all production runs to monitor the accuracy of the HDNNP for all configurations visited during our extensive MD simulations.

### Neural Network Parametrizations

The HDNNPs used in this work were constructed and trained with the n2p2 package.^45^ In total, 50 radial and angular polynomial symmetry functions (p2 type) were used with a cutoff radius of *r*_c_ = 7 Å. The parameters of the symmetry functions are listed in Table S1 of Supporting Information. The atomic neural networks have a topology of 50-60-1 and employ the softplus function as the first activation function and the linear function for the output layer.

We have trained a committee of eight HDNNPs on a reference data set of 5342 structures, containing 138, 139, and 140 atoms. Each of the eight committee members was initialized with different weights and biases, and a randomly chosen 10% of the data set was used as a validation set. The committee of HDNNPs reproduces the energies and forces of the training set with a root mean squared error of 2.25 eV and 45.5 meV/Å, respectively. Parity plots for energy and forces can be found in the Supporting Information.

The data set and the trained models are available on the public repository Zenodo.^46^

### Molecular Dynamics Simulation and Analysis

All HDNNP-MD simulations were performed using the large-scale atomic/molecular massively parallel simulator (LAMMPS).^47^ We simulated at three temperatures, namely 304, 330, and 359 K, to determine the temperature-dependent properties of the monovacancy. For each temperature, we carried out two independent MD runs. They add up to 0.4 μs of simulation time overall for 304 and 330 K and 0.3 μs for 359 K. Each simulation was first equilibrated for 1 ns in the canonical ensemble (*NVT*) with the Langevin thermostat, followed by a run in the microcanonical ensemble (*NVE*) for the remaining simulation time (0.15–0.2 μs). We employed the velocity Verlet integrator with a time step of 1 fs. Only the *NVE* segments of the simulations were used to determine the hopping rates. Diffusion coefficients were determined from the mean square displacement of the defects according to the Einstein formula using the MD analysis code.^48,49^ A simulation time of 9 ns/day has been realized utilizing 4 cores (AMD Ryzen 9 5950X CPU) for a supercell containing 139 atoms.

## Results

**Figure 1:**
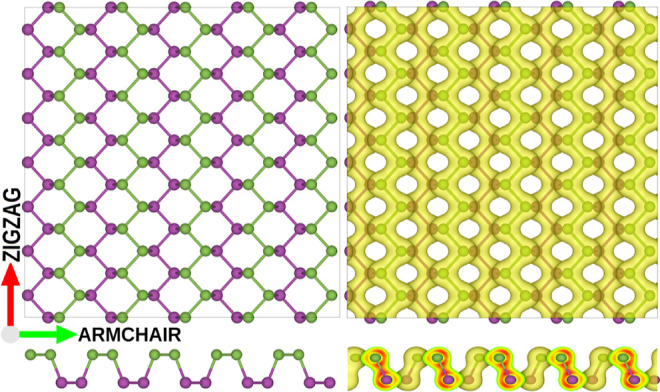
Left panel: Phosphorene supercell employed for our calculations in top and side view. Atoms are colored green and purple according to the sublattice to which they belong. The zigzag and armchair directions are indicated by arrows. Right panel: Same structure as on the left, with an isosurface of the total electronic charge distribution superimposed, again in the top and side views. The colors indicate different values of the electronic charge density.

Black phosphorene exhibits a nonplanar honeycomb lattice comprising two sublattices. Phosphorus has a 3s^2^3p^3^ electronic configuration, and each atom forms three covalent bonds with its nearest neighbor, one of which lies on a different sublayer, as illustrated in Figure 1.

### Monovacancy

**Figure 2:**
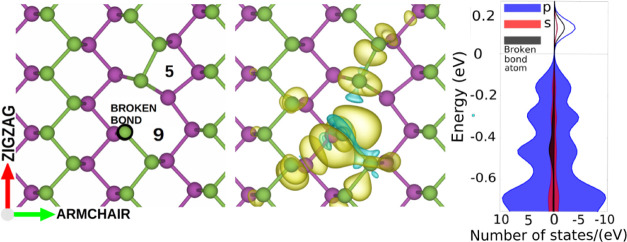
Left: Single vacancy SV(5|9) was obtained by removing one atom from the top layer (green) and relaxing the energy. The black circle indicates the atom that takes part in only two bonds. Center: Difference between the total electronic charge densities of the spin-up (yellow) and spin-down components (cyan). Right: The electronic density of states and the positive and negative values represent the spin-up and spin-down components, respectively.

The single-vacancy defective structure SV(5|9), shown in Figure 2, is obtained by removing one atom from the pristine structure and relaxing it. Here, the notation (5|9) refers to the two distorted 5- and 9-membered rings of atoms comprising the vacancy. According to our DFT calculations, the SV(5|9) defect has a formation energy of 1.65 eV and a total magnetic moment of 1 μ_Bm_, in good agreement with previous results.^18,23,28,50−54^ The magnetism arises from a dangling bond atom, which forms only two bonds instead of three. Consequently, there is an unpaired electron, mainly localized in the vicinity of the broken bond (see Figure 2). The density of states shows an occupied peak above the Fermi energy near the bottom of the valence band. This corresponds to a defect state and represents the possibility of the atom with a broken bond hosting another electron.

### Mechanisms of Diffusion

**Figure 3:**
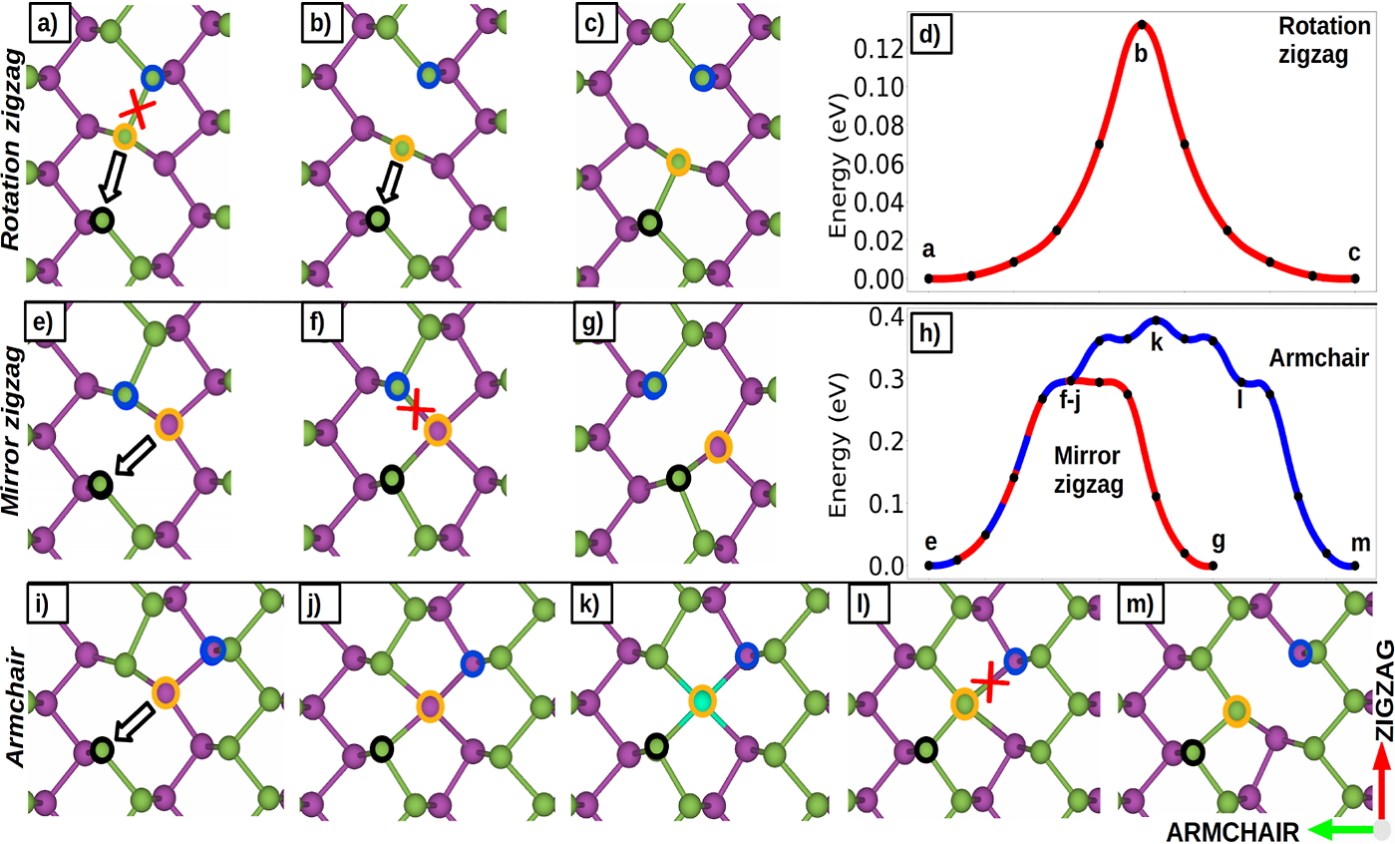
Mechanisms of diffusion of the monovacancy and the corresponding ciNEB energy path. The black circle surrounds the atom with an initial unsaturated bond, whereas the yellow one indicates the atom that breaks a bond during the process, and the blue circle is where the vacancy ends up in the transition. (a–d) *Rotation* zigzag. (e–h) *Mirror* zigzag. (i–m) *Armchair*. The *mirror* and *armchair* transitions undergo the same mechanism until the intermediate state f, where a four-folded atom is formed.

By performing DFT-MD calculations at 300 K, we have observed transitions of the monovacancy between different sites along the zigzag direction. We call this transition a *rotation* since the final defect configuration can be obtained by applying a 180° rotation to the initial one. The mechanism for this transition is shown in Figure 3a–c. The black-circled atom has one unpaired electron, and to saturate its p shell, it attracts the yellow one. This leads to the breaking of the bond between the blue and yellow atoms. This hopping event can occur back and forth multiple times. At the transition state (b), there is a competition between the black and the blue atoms, which are exerting an attractive force on the yellow one. The estimated energy barrier for this process is 0.13 eV, as inferred from the energy profile calculated with ciNEB and displayed in Figure 3d.

Two further types of vacancy transitions are possible toward different sites, which exhibit higher energy barriers compared to the *rotation*. Observing them in ab initio MD simulations would require prohibitively long simulation times. However, starting with the preadapted configuration obtained from the HDNNP-MD allowed us to correctly model and reproduce these transitions with first-principle accuracy.

The so-called *mirror* transition, shown in Figure 3e–g, occurs along the zigzag direction as well, and the orientation of the final configuration can be obtained by applying a *mirror* operation to the initial one. The estimated ciNEB energy barrier is 0.3 eV, as indicated in Figure 3h. At the transition state, one phosphorus atom forms four bonds (Figure 3f). Starting from this configuration, a transition can occur following two distinct mechanisms: 3e–g, as we just discussed, and 3i–m. In the latter, the four-folded atom can move along the direction perpendicular to the sheet to the other atomic sublayer, Figure 3j. The associated energy barrier is estimated to be 0.1 eV. From here, the monovacancy can hop to two different sites along the armchair direction; Figure 3k shows one possible transition state. Therefore, the overall process has an energy barrier of 0.4 eV (3l). This diffusion mechanism is referred to as the *armchair* and is the only route for the monovacancy to switch from one sublayer to the other. Table 1 provides an overview and comparison of energy barriers with those of other works. Movies S2, S3, and S4 showing all three types of hopping mechanisms are available in the Supporting Information.

**Table 1:** Energy Barriers for Monovacancy Diffusion Obtained by ciNEB and Derived from HDNNP-MD (as Explained in the Section Kinetic Properties), Together with Values Reported in Other Works

energy barrier (eV)	*rotation*	*mirror*	*armchair*
NEB	0.13	0.30	0.39
HDNNP-MD	0.09	0.25	
Hu^18^			0.40
Hu^51^	0.18		0.38
Cai^28^	0.18	0.30	0.40
Li^52^			0.40
Vierimaa^23^	0.09	0.25	
Gaberle^53^	0.25	0.44	0.57
Rijan^54^		0.31	

As a result, the combination of these three transition mechanisms allows the SV(5|9) to diffuse along every direction of the lattice across the whole monolayer.

### Monovacancy Tracking

**Figure 4:**
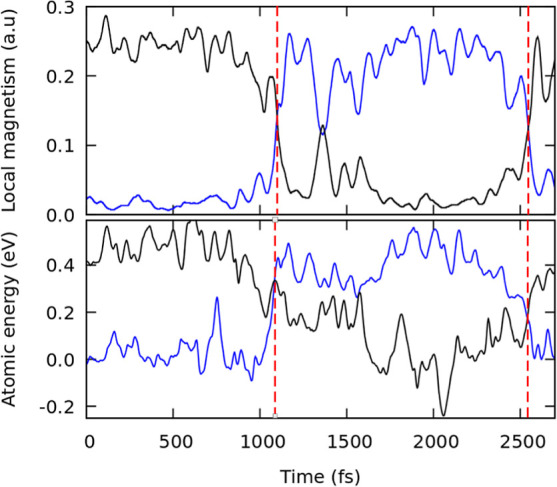
Monovacancy hopping can be detected by changes in either the local magnetic moments for DFT-MD (top) or the atomic energies for HDNNP-MD (bottom). The blue and black lines show the local magnetic moments and atomic energies for the two atoms involved in the hopping of the defect. Both methods predict hopping at the same time on the same trajectory. The average atomic energy is subtracted from the atomic energies.

Keeping track of the location of the monovacancy is necessary to assess its kinetic properties. This can be easily done for DFT-MD because of the local magnetic moment, which clearly distinguishes an atom with an unsaturated bond. For instance, Figure 4 (top) shows two *rotation* hopping events between two atoms and the corresponding exchange of local magnetic moments.

Within HDNNP-based simulations, the magnetic moment is not accessible, and visual detection of monovacancy positions in long MD consisting of millions of timesteps is impractical. Here, we detect the position of the monovacancy by exploiting a particular property of HDNNPs with minimal computational overhead. In HDNNPs, the total energy is calculated as the sum of local atomic energies (see eq 1). When a monovacancy is introduced into a material, its formation energy is spread around the monovacancy. Most of the formation energy is attributed to the atom with an unsaturated bond. By simply finding the atom with the highest atomic energy, we identified the position of the monovacancy in the material for any given configuration. A hopping process is then signaled by a change in the index of the atom with the highest energy, and the type of transition can be inferred from the relative positions of the new and old atoms.

Figure 4 compares the two techniques for the same MD trajectory. Atomic energy exchanges between atoms in the HDNNP-MD occur precisely at the same time as local magnetic moment swaps in the DFT-MD, validating our new approach.

### Kinetic Properties

Although energy barriers offer some insights into the kinetics of monovacancies, they are insufficient for the estimation of hopping rates ν due to the unknown pre-exponential factor ν_s_, also called the characteristic frequency, in the Arrhenius equation
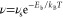
6

**Figure 5:**
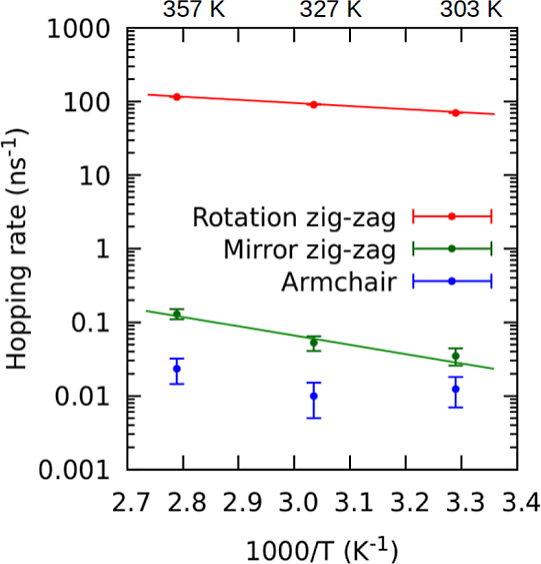
Arrhenius plot of the hopping rates for each of the three diffusion mechanisms at 304, 330, and 359 K, as determined by HDNNP-MD.

The pre-exponential factor ν_s_ can be estimated in the harmonic approximation from the atomic vibrational frequencies in the energy minimum and the transition state associated with the motion of the MV using the Vineyard formula.^55^ The hopping rate can also be obtained directly from an MD simulation without resorting to the Arrhenius equation. If a given type of hopping mechanism is observed many times during an MD simulation, then the hopping rate is simply given by the number of occurrences divided by the total simulation time. The hopping rates estimated directly from HDNNP-MD are shown in Figure 5. The reported values at room temperature for zigzag *rotation*, zigzag *mirror*, and *armchair* hopping rates are 71, 0.035, and 0.013 ns^–1^, respectively.

Although we also report highly itinerant atomic vacancies as in previous work,^28^ a quantitative agreement in hopping rates is found only for the *mirror* transition. Our hopping rates are higher by 3 orders of magnitude for the *armchair* transition and 1 order of magnitude for the *rotation* transition. To reveal the cause of this discrepancy, we have factorized the hopping rates via the Arrhenius equation (eq 6) into the pre-exponential factor (Table 2) and the exponential factor depending on the energy barrier (Table 1). For the *rotation* mechanism, the prefactors agree very well. The difference in hopping rates comes from the energy barrier, which is significantly smaller in our work (0.09 vs 0.18 eV). The previously reported prefactor for the *mirror* mechanism is an order of magnitude larger than ours but also accompanied by a higher energy barrier (0.25 vs 0.30 eV). These two effects compensate for each other, leading to similar hopping rates. Insufficient statistics prevent us from estimating the activation energy and factorizing the rate for the *armchair* transition, such that the discrepancy cannot be explained in this case.

**Table 2:** Hopping Rates at 300 K and Pre-exponential Factors for MV Diffusion Derived from HDNNP-MP Compared with Previous Work^28^^a^

	*rotation*		*mirror*		*armchair*	
	ν	ν_s_	ν	ν_s_	ν	ν_s_
HDNNP	71	1800	0.035	180	0.013	
Cai^28^	2.5	2200	0.021	2400	3.1 × 10^–5^	160

a All numbers are given in units of ns^–1^.

According to our results, the diffusion coefficient of a monovacancy in the in-plane direction is significantly greater than the diffusion coefficients of a divacancy and a tetravacancy. We determined the diffusion coefficient for the monovacancy to be 10^–11^ m^2^/s at 359 K. Experimental measurements report the diffusion coefficients for divacancy and tetravacancy to be 10^–19^ to 10^–20^ m^2^/s at 573 K.^26^ This difference can be attributed to the size of the diffusion barrier, which is considerably lower for monovacancy, ranging from 0.1 to 0.4 eV, compared to divacancy and tetravacancy, which exhibit higher diffusion barriers in the range of 2.1–3.0 eV.^26^

**Figure 6:**
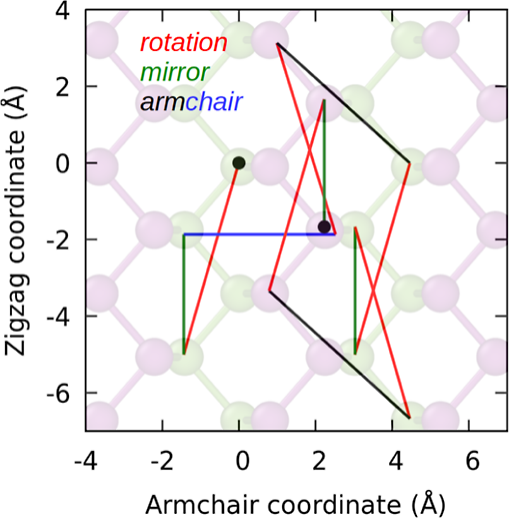
Trajectory of monovacancy diffusion during a period of 200 ns at 328 K, as simulated by HDNNP-MD. Each color corresponds to a specific mechanism type, with *rotation* represented by red, *mirror* by green, and the two types of *armchair* transition denoted by blue and black.

A diffusion trajectory of the monovacancy at 328 K over a 200 ns simulation time is presented in Figure 6. The monovacancy started at the origin and diffused through a combination of different mechanisms. Its maximum diffusion distance from the origin was 8 Å, but after 200 ns, the distance was only 3 Å. Within this time frame, the monovacancy underwent 18 × 10^3^*rotations*, 11 *mirrors*, and 3 *armchair* transitions. Only 5 *rotations* and 3 *mirror* events resulted in true displacements of the vacancy, as the remaining ones were negated by back hops. In contrast, all *armchair* transitions led to true displacements.

### Divacancy and Coalescence of Monovacancies

A divacancy is created by removing two atoms from the pristine structure. Since, in this case, all bonds are fully saturated, there is no magnetism overall. The most stable divacancy has a formation energy of about 1.36 eV,^28^ which is significantly lower than the 3.3 eV formation energy of two separated SV(5|9). Such a large energy difference, along with high monovacancy mobility, implies that monovacancies tend to coalesce quickly. However, the coalescence of two monovacancies into one divacancy has not yet been documented.

**Figure 7:**
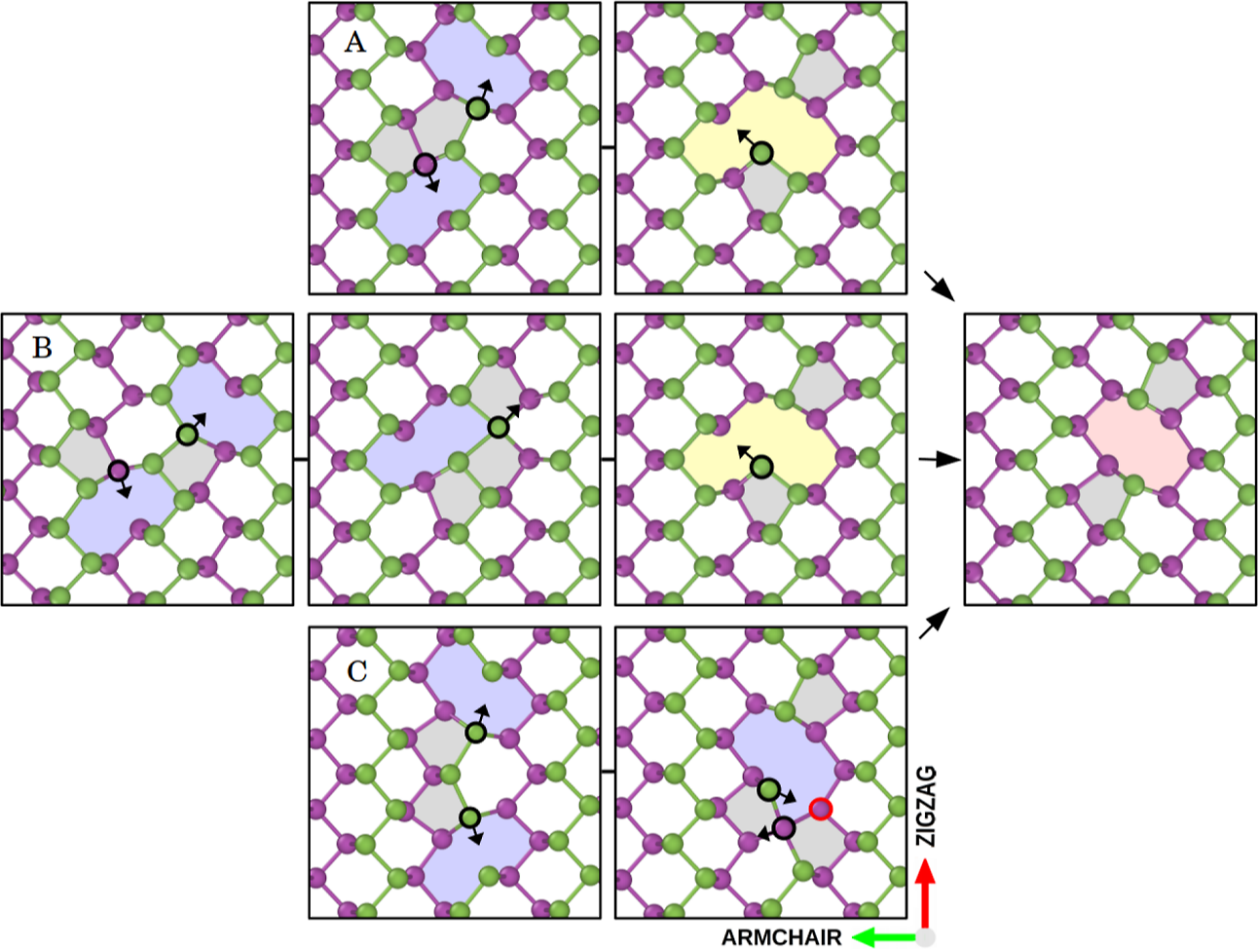
Divacancy formation mechanisms. Initial configurations of the two monovacancies that eventually coalescence to form DV(5|8|5) are represented by the letters A, B, and C. Transitions A and B share the same last intermediate state before coalescence. The black arrows indicate the future direction of the atomic movement. Colored areas indicate rings formed by more or less than six atoms.

Monovacancies can approach one another from different directions and with different configurations, leading to various types of mechanisms and divacancy conformations. Because of the complexity of the mechanism, MD is the appropriate method for studying vacancy coalescence. Since there is no prior knowledge of the coalescence of monovacancies, we introduce 50 initial arrangements differing in both the distance and mutual orientation of the vacancies and let the system evolve with HDNNP-MD. In this process, many new structures were added to the reference data set for training the HDNNP. Although we found many metastable states with formation energies of around 2.5 eV, our interest is in coalescence into stable divacancies with formation energies of 1.3–1.4 eV. Figure 7 shows the three initial arrangements of monovacancies and mechanisms of coalescence into DV(5|8|5) with a 1.4 eV formation energy. Movies S5, S6, and S7 in Supporting Information provide additional support for this visualization and analysis. All three mechanisms have been identified by HDNNP and validated by DFT. The mutual orientation of monovacancies is identical except for the relative position in the armchair direction. This finding is crucial because diffusion is less likely to occur in this orientation than in a zigzag.

Transitions A and B share the last intermediate state before coalescence, but they go through different transition states in the initial stage. Mechanism A involves only two *rotation* transitions. In contrast, mechanism B needs the *armchair* transition in addition to the *rotation* transition. Two *rotations* are also necessary in the C transition. Based on the hopping sequence of the atoms, one of the black-labeled atoms forms four bonds, and the second one is left with two bonds. The divacancy is finally obtained when the four-bonded atom breaks the bond with the black atom, and the bond is formed with the other red atom.

The coalescence mechanism A is the fastest because only the *rotation* barrier, at 0.13 eV, must be crossed. Coalescence C initially only needs a *rotation*, but it takes a longer time than A because of the bond exchange of the black atoms with the red atom. Nevertheless, the process can be observed in a few ns. Since it requires an *armchair* transition with a 0.4 eV energy barrier, coalescence B proceeds the slowest of all.

The resulting DV(5|8|5) in Figure 7 was experimentally observed as the most stable DV^26^, even though previous computational studies indicate that DV(5|7|7|5) should be more stable.^26,28^ Yao *et al.* attribute this to nonequilibrium conditions brought on by energetic e-beam irradiation.^26^ While the relative stability of DV(5|8|5) and DV(5|7|7|5) has not been resolved, the transition mechanisms between these two double vacancies and DV diffusion have been analyzed theoretically and experimentally.^26^

**Figure 8:**
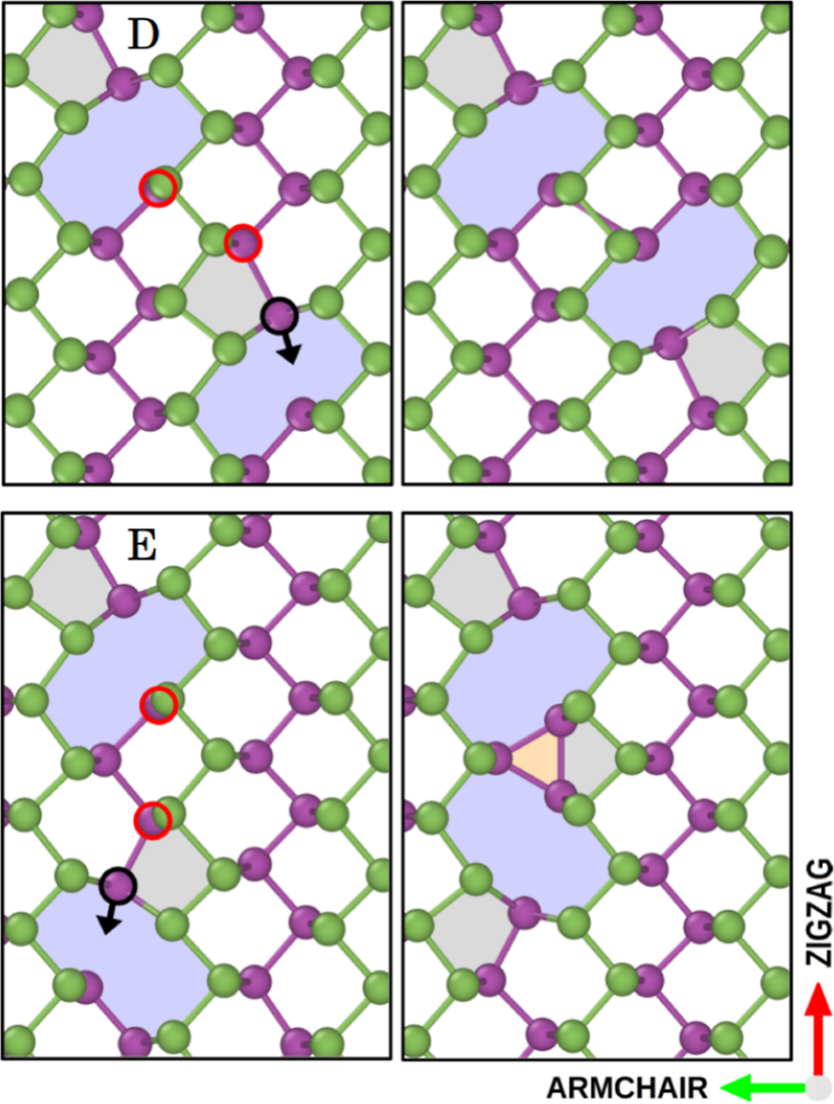
Metastable monovacancy pair formation mechanisms. MV pairs are formed if atoms with broken bonds (red circles) are close enough to make a bond. Mechanism E has also been reported in previous work.^56^

The initial orientation of the atoms with dangling bonds plays an important role in the coalescence process. When these atoms are close to one another, they instantly form a bond that hinders further evolution into more stable divacancies. Two examples of this mechanism are shown in Figure 8. Only *rotation* transitions of black atoms bring the two bonded red atoms together, which causes a bond to form right away. These states could be thought of as MV pairs rather than divacancies since they maintain MV shapes. Their formation energy, 2.3 eV, falls exactly between the stable divacancy (1.4 eV) and the two separated monovacancies (3.3 eV).

## Conclusions

In this paper, we thoroughly analyze the mobility of phosphorene monovacancy. All three of the previously identified diffusion mechanisms have been successfully reproduced by both DFT-MD and HDNNP-MD. The energy barriers calculated statically from DFT with the nudged elastic band method and dynamically from HDNNP-MD are in good agreement with each other. MD simulations longer than 1 ms in total have not revealed any novel mechanisms. By assessing hopping rates directly from MD, we confirm the highly itinerant nature of the phosphorene monovacancy even at room temperature, preferentially along the zigzag direction.

For the coalescence of two monovacancies into one divacancy, we identified three mechanisms, resulting in the stable DV(5|8|5) conformation with a formation energy of 1.4 eV. Coalescence can be observed on the nanosecond time scale if MVs are close enough and ideally oriented. However, if the atoms with the dangling bond approach one another too early, a metastable MV pair rather than a fully coalesced DV is produced, emphasizing the significance of the initial configuration. Monovacancies are predicted to be the most common defect after electron and ion irradiation. But since they quickly coalesce, their potential utilization poses a challenge, suggesting turning the attention to more stable divacancies.

## Supporting Information Available

S1: Parameters of the radial and angular symmetry functions for phosphorene and parity plots comparing predicted values from an ensemble of HDNNPs with a size of 8 against target values obtained by DFT (PDF)
S2: Movie of the rotation mechanism shown in Figure 3a–c (MP4)
S3: Movie of the mirror mechanism shown in Figure 3e–g (MP4)
S4: Movie of the armchair mechanism shown in Figure 3i–m (MP4)
S5: Movie of the coalescence mechanism shown in Figure 7A (MP4)
S6: Movie of the coalescence mechanism shown in Figure 7B (MP4)
S7: Movie of the coalescence mechanism shown in Figure 7C (MP4)
