# Supplementary material for: Diffusion and Coalescence of Phosphorene Monovacancies Studied Using High-Dimensional Neural Network Potentials
Source: J Phys Chem C Nanomater Interfaces. 2023 Dec 5;127(49):23743–51. doi: 10.1021/acs.jpcc.3c05713 (PMC10726346; doi:10.1021/acs.jpcc.3c05713)
Supplement: Supplementary file 1 — jp3c05713_si_001.pdf [file jp3c05713_si_001.pdf]

Supporting Information for Publication: Diffusion and Coalescence of Phosphorene Monovacancies studied using High-Dimensional Neural Network Potentials

Lukáš Kývala, Andrea Angeletti, Cesare Franchini, and Christoph Dellago
University of Vienna, 1090 Vienna, Austria

Table S1: Parameters of the radial and angular symmetry functions for phosphorene.

	r_{min} (Å)	r_{max} (Å)	θ_{min} (°)	θ_{max} (°)
G^{rad}	-7	7	-	-
	-6	6	-	-
	-5	5	-	-
	-4	4	-	-
	-3	3	-	-
	3	7	-	-
	2	6	-	-
	1	5	-	-
	0	4	-	-
	-1	3	-	-
G^{ang}	-7	7	-180	180
	-6	6	-180	180
	-5	5	-180	180
	-4	4	-180	180
	-3	3	-180	180
	3	7	-180	180
	2	6	-180	180
	1	5	-180	180
	0	4	-180	180
	-1	3	-180	180
	-7	7	0	180
	-6	6	0	180
	-5	5	0	180
	-4	4	0	180
	-3	3	0	180
	3	7	0	180
	2	6	0	180
	1	5	0	180
	0	4	0	180
	-1	3	0	180
	-7	7	0	90
	-6	6	0	90
	-5	5	0	90
	-4	4	0	90
	-3	3	0	90
	3	7	0	90
	2	6	0	90
	1	5	0	90
	0	4	0	90
	-1	3	0	90
	-7	7	90	180
	-6	6	90	180
	-5	5	90	180
	-4	4	90	180
	-3	3	90	180
	3	7	90	180
	2	6	90	180
	1	5	90	180
	0	4	90	180
	-1	3	90	180

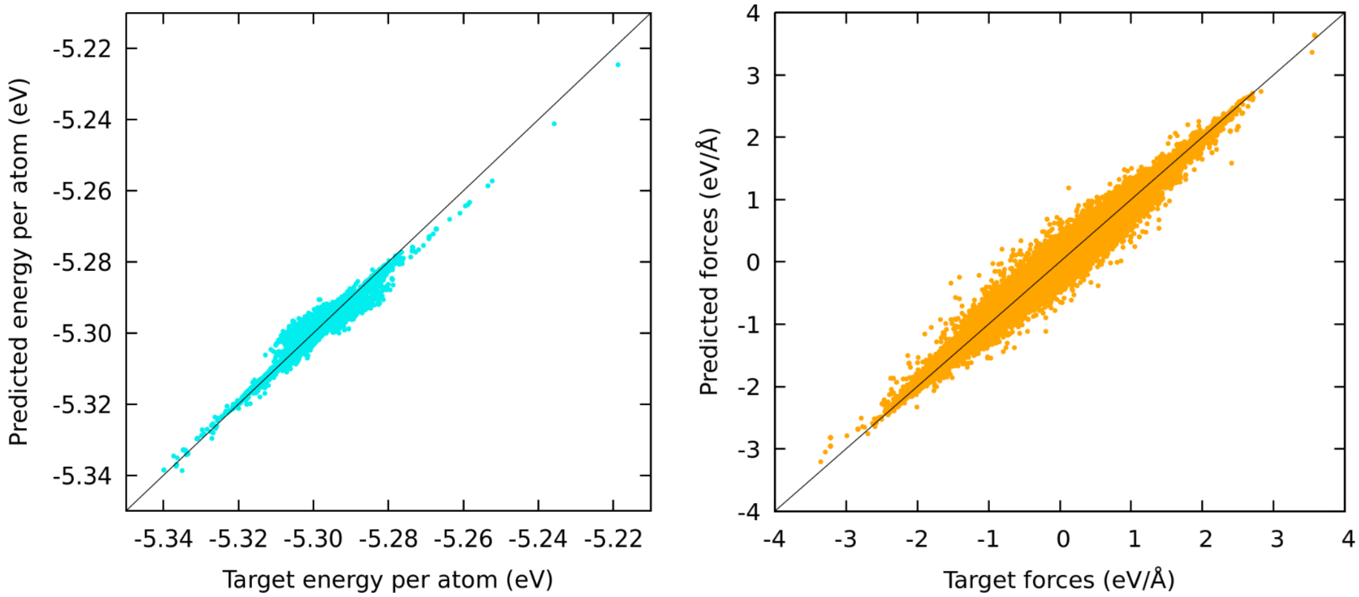

Figure S1: Parity plots comparing predicted values from an ensemble of HDNNPs with a size of 8 against target values obtained through DFT.
